# Simulated Discharge of Ballast Water Reveals Potential Contribution to Spread of Antibiotic Resistance Genes in Geographically Isolated Receiving Waters

**DOI:** 10.3390/antibiotics14040340

**Published:** 2025-03-26

**Authors:** Jianhong Shi, Chengyuan Ji, Rui Wang, Chaoli Sun, Baoyi Lv

**Affiliations:** 1 College of Ocean Science and Engineering, Shanghai Maritime University, Shanghai 201306, China; 2 CCCC National Engineering Research Center of Dredging and Equipment Co., Ltd., Shanghai 200082, China; 3 Key Laboratory of Dredging Technology, CCCC, Shanghai 200082, China

**Keywords:** ARG transmission, bacterial community, marine environment, ecological risk

## Abstract

**Background/Objectives:** The propagation of antibiotic resistance genes (ARGs) poses a huge threat to environmental and human health. The ballast water from ships has been recognized as an important vector of ARGs. However, little is known about how ballast water from geographically isolated water affects ARGs in receiving waters. **Methods:** Herein, we investigated the changes in ARGs in receiving water by microcosm experiments simulating the discharge of ballast water. **Results:** The simulated discharge of ballast water increased the abundances of target ARGs, which were 1.3–5.6-fold higher in the mixture of ballast water and receiving water (microcosm M) than in receiving water at the end of the experiment. The enrichment of target ARGs was significantly associated with MGEs. Moreover, the discharge of ballast water changed the microbial communities in receiving water. Further network analysis identified potential ARG hosts, such as *Pseudohongiella*a and *Amphritea*, with the abundance in microcosm M (0.23% and 0.036%) being higher than in receiving water (0.09% and 0.006%), the changes of which might be responsible for ARG variations. **Conclusions:** Overall, our findings suggest the discharge of ballast water might promote the spread of ARGs in different geographical waters and the corresponding ecological risks should not be ignored.

## 1. Introduction

Antibiotics are widely used in human medicine and aquaculture to treat bacterial infections and diseases [1]. Many residual antibiotics have entered the environment, resulting in the dissemination and enrichment of antibiotic resistance genes (ARGs) [2,3]. ARGs are now recognized as an emerging pollutant as they can spread rapidly in the environment through horizontal gene transfer mediated by mobile genetic elements (MGEs) [4,5]. The prevalence and dissemination of ARGs have become global problems for environmental systems and public health. At present, ARGs have been largely detected in aquatic environments, including rivers, lakes, and seawater. Several studies have reported that the discharge of wastewater and aquaculture waste could accelerate the enrichment of ARGs downstream or in the nearby waters [6,7]. Therefore, it was critical to understand the effect of the introduction or discharge of exogenous water on ARGs in the receiving water to control the development of antibiotic resistance.

Shipping is the main means of freight transport and accounts for more than 90% of the global freight volume. Ballast water is necessary for safe navigation as it maintains a ship’s balance [8]. Nearly 10 billion tons of ballast water are transferred annually worldwide [9]. Ballast water is a considerable pathway of biological invasion as it contains harmful algae, bacterial pathogens, and viruses [10,11,12,13,14]. Notably, the ballast water has become the hotpot of ARGs. In total, 26 types and 710 subtypes of ARGs were detected in ballast water from 13 ships from 11 countries and regions [15], which was more diverse than that in some natural water, such as the Pearl River [16] and sixteen estuaries in China [17]. The abundance of tetracycline ARGs in ballast water was even higher than that in ocean water [18]. Predictably, ballast water should be regarded as the potential hotpot for the transmission of ARGs across geographically isolated waters.

It was well known that the ecological microflora of the incoming ships’ ballast and receiving water bodies are obviously different as the departure and destination ports of foreign-going vessels are always far from each other [19,20,21]. Previous studies reported that the presence of ARGs was strongly linked to bacterial composition because of bacteria acting as important hosts of ARGs [22]. When the ballast water enters the receiving water, the species that are unable to adapt to the new environment may be eliminated while some species may become dominant as they are able to survive and grow [11,23]. As a result, it is largely unknown whether and how the bacterial composition and ARG hosts can be changed; thus, it is unknown how the inclusion of ballast water affects the ARGs in the receiving waters.

In this study, the discharge of ballast water was simulated by performing ballast water/receiving water microcosm experiments. The variation in ARGs/MGEs and bacterial communities in the microcosms was determined. The aims of this study were to (i) investigate the effects of ballast water discharge on the abundance of ARGs in receiving waters and (ii) explore the shift in MGEs and bacterial communities and their relationships with ARG variation in receiving waters. To our knowledge, this study provides the first evidence of the dissemination of ARGs in receiving waters that are affected by ballast water.

## 2. Results and Discussion

### 2.1. ARG Profiles in Initial Samples

**Figure 1:**
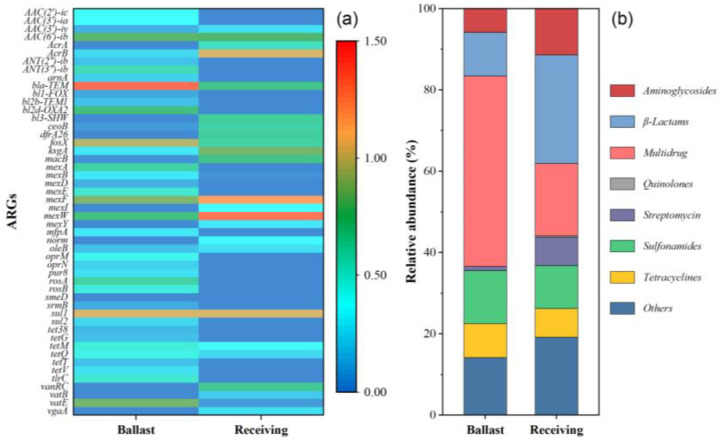
Abundance of the ARG subtypes (**a**) and relative percentages of ARG types (**b**) in initial ballast and receiving water.

A total of 41 and 25 subtypes of ARGs were annotated for the ballast and receiving water samples, respectively (Figure 1a, Table S3). The top 10 most abundant ARGs included *bac*A, *mex*W, *mex*F, *sul*1, *fos*X, *acr*B, *vat*E, *aac*(6″)-ib, *ksg*A, and *aac*(3″)-ia. These ARG subtypes belonged to the class of sulfonamides, tetracyclines, β-lactams, aminoglycosides, streptomycins, quinolones, multidrug, and other resistance genes, and their percentages were 13.0% and 10.5%; 8.4% and 7.1%; 10.7% and 26.6%; 5.9% and 11.4%; 1.5% and 7.0%; 0% and 0.3%; 46.8% and 17.8%; and 13.7% and 19.1% in the ballast and receiving waters, respectively (Figure 1b). These results indicated that diverse and abundant ARGs exist in these two samples, especially the ballast waters. It is well known that most of the ballast water comes from the inshore waters, which are susceptible to contamination by antibiotics from aquaculture and sewage [2,24]. Moreover, the profile of ARGs was obviously different between the ballast and receiving waters. The relative abundance of sulfonamides, tetracyclines, and multidrug resistance genes was higher in ballast water compared to receiving water, while the aminoglycosides, β-lactams, and streptomycins resistance genes showed the opposite trend. Those results suggested the potential risk of ARG dissemination through ballast water.

### 2.2. Changes in ARGs and MGEs in Microcosms

In order to further understand the effects of ballast water on ARGs in receiving water, several target ARGs that dominated in the four ARG classes (sulfonamides, tetracyclines, β-lactams, and aminoglycosides) were quantified in the initial ballast and receiving water, in addition to three microcosms by qPCR. The abundance of *sul*1, *tet*M, *tet*Q, and *bla*_TEM_ was 1.7 × 10^−4^ and 1.2 × 10^−5^; 1.8 × 10^−6^ and 9.1 × 10^−7^; 3.3 × 10^−6^ and 2.1 × 10^−6^; and 3.6 × 10^−7^ and 1.2 × 10^−7^ copies/16S rRNA in the initial ballast and receiving water samples, respectively. The gene *sul*1 had the highest abundance among the detected ARGs, in accordance with previous reports of high abundances of *sul*1 in various types of water bodies, including aquaculture, lakes, groundwater, and seawater [25,26]. The genes *tet*M and *tet*Q widely exist in marine environments and can be transferred among bacteria [27,28]. Notably, the abundances of target genes, especially for *sul*1 *and bla*_TEM_, were obviously higher in the ballast water than in the receiving water (*p* < 0.05), which might be related to the heavy metal pollution in the ballast tank [29,30]. Heavy metal has been considered an important co-selection agent for ARGs [31]. Moreover, horizontal gene transfer is driven by MGEs, which could increase the spread of ARGs [22]. Therefore, MGEs comprising *intI*1 and *intI*2 were detected in the initial samples. The abundances of *intI*1 and *intI*2 were 1.3 × 10^−5^ and 3.0 × 10^−6^ copies/16S rRNA and 2.2 × 10^−5^ and 3.0 × 10^−6^ copies/16S rRNA in the initial ballast and receiving water samples, respectively.

**Figure 2:**
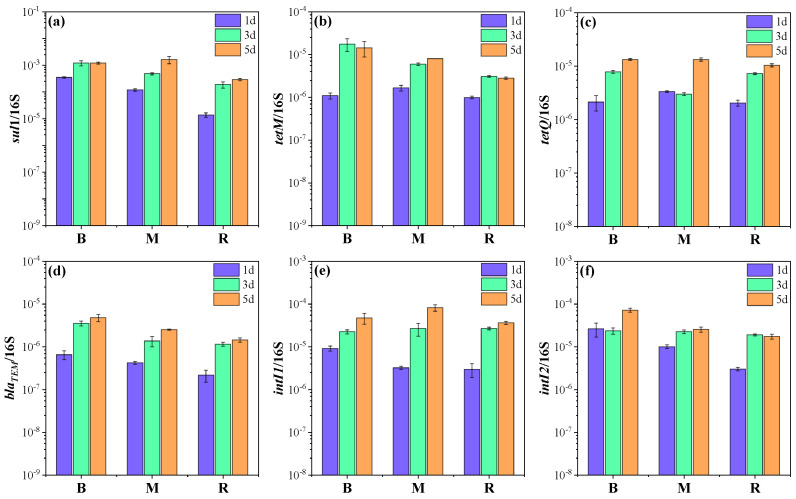
The variation in ARG and MGE abundances with the culture time in each microcosm: (**a**) *sul*1; (**b**) *tet*M; (**c**) *tet*Q; (**d**) *bla*_TEM_; (**e**) *intI*1; (**f**) *intI*2. Microcosm B (ballast water), microcosm M (ballast water and receiving water at a ratio of 1:1), and microcosm R (receiving water).

All target ARGs exhibited similar trends in the microcosms, and their abundances in microcosm M were higher than that in microcosm R (Figure 2, Table S4). Specifically, the abundance of *sul*1 in microcosm M was 1.2 × 10^−4^, 4.9 × 10^−4,^ and 1.6 × 10^−3^ copies/16S rRNA on days 1, 3, and 5, which was 2.6–8.7-fold higher relative to that in microcosm R (*p* < 0.05). It was worth noting that *sul*1 was even more abundant in microcosm M than in microcosm B on day 5. For *tet*M, the abundance in microcosm M was 1.7 × 10^−7^, 6.0 × 10^−7,^ and 8.1 × 10^−7^ copies/16S rRNA on days 1, 3, and 5, which was 1.7-, 1.9-, and 2.9-fold higher relative to that in microcosm R and lower than that in microcosm B. The abundances of *tet*Q and *bla*_TEM_ decreased in the order of microcosm B > M > R and were 1.3- and 1.8-fold higher in microcosm M than in microcosm R at the end of the experiment. These results indicated that the discharge of ballast water could contribute to the enrichment of ARGs in the receiving water. The findings were similar to those of previous studies that effluents from wastewater treatment plants could evidently elevate the ARG level in natural waters [6,32]. Among target ARGs, *sul*1 exhibited the greatest enrichment potential with the introduction of ballast water, which was probably associated with its high horizontal transfer potential. It has been frequently reported that *sul*1 can be carried by class 1 integron, which is a significant player in the horizontal transfer and global spread of ARGs [33,34]. Moreover, previous studies have also found that the wastewater discharge led to an increased abundance of *tet*M and *tet*Q in the receiving river [32,35].

In addition, the MGEs in microcosm M were more abundant than those in microcosm R during the experiment. The abundance of *intI*1 and *intI*2 in microcosm M was 8.2 × 10^−5^ and 2.6 × 10^−5^ copies/16S rRNA on day 5, which was 2.2- and 1.5-fold higher than that in microcosm R (Figure 2e,f). Interestingly, *intI*1 was 1.7-fold higher in microcosm M than in microcosm B. The results suggest that ballast water increased the level of MGEs in receiving water as well. It should be noted that MGEs can carry ARGs and can be transferred between homogeneous and even heterogeneous bacteria, thereby promoting the spread of ARGs [22]. Zhang et al. reported that *intI*1 could be used as an indicator of the potential of ARG dissemination [36]. As shown in Table 1, a significant positive correlation was found between *intI*1 and *sul*1, *bla*_TEM_. Moreover, *intI*2 had a positive relationship with *tet*M, *tet*Q, and *bla*_TEM_. Considering the more abundant ARGs and MGEs in ballast water than in receiving water and especially the higher abundance of *sul*1 and *intI*1 in microcosm M than in microcosm B, it is likely that horizontal transfer would occur in the receiving water. Therefore, the ecological risks associated with the higher abundance of ARGs in ballast water should not be ignored as ARGs may be propagated by horizontal gene transfer.

### 2.3. Transformation of Bacterial Communities in Microcosms

**Figure 3:**
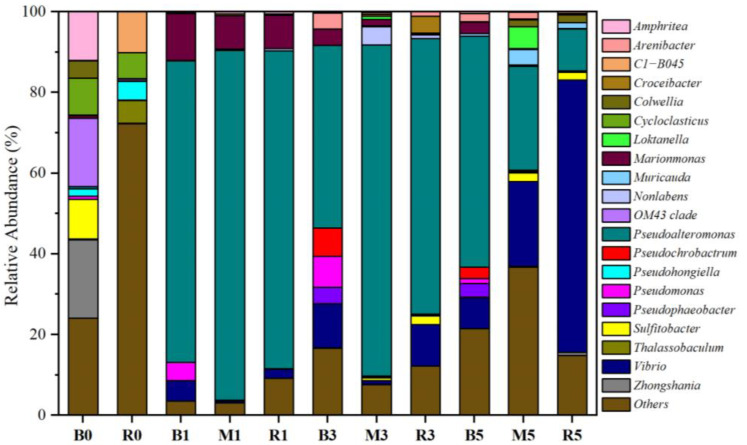
The relative abundance variation in taxonomic genus with the culture time in each microcosm. Microcosm B (ballast water), microcosm M (ballast water and receiving water at a ratio of 1:1), and microcosm R (receiving water). B0 and R0 indicate the initial ballast and receiving water, respectively. B1, B3, and B5 indicate the microcosm B on days 1, 3, and 5, respectively. R1, R3, and R5 indicate the microcosm R on days 1, 3, and 5, respectively. M1, M3, and M5 indicate the microcosm M on days 1, 3, and 5, respectively.

The bacterial communities were analyzed in the initial ballast and receiving water, as well as three microcosms. The dominant phylum exhibited obvious differences in the initial ballast and receiving waters (Figure S1). Proteobacteria was the predominant phylum in ballast water, accounting for over 95% of the total community, while Proteobacteria (67.5%), Bacteroidetes (12.5%), Chloroflexi (4.9%), Actinobacteria (3.6%), and Patescibacteria (2.3%) were prevalent in the receiving water. The result was consistent with previous studies that the dominant phyla in seawater were Proteobacteria, Bacteroides, and Actinobacteria [2,13]. Moreover, clear differences were also observed in the bacterial community between the initial ballast and receiving waters at the genus level (Figure 3). *Sulfitobacter* (9.8%), *OM43 clade* (16.9%), *Zhongshania* (19.5%), and *Amphritea* (12.1%) were dominant genera in ballast water, while *Thalassobaculum* (5.6%), *C1-B405* (10.1%), and *Pseudohongiella* (4.7%) occupied the majority of genera in the receiving water.

Proteobacteria and Bacteroidetes were the dominant phyla and accounted for more than 99% of the total sequences in three microcosms (Figure S1). The relative abundance of Bacteroidetes gradually increased from 0.8% on day 1 to 11.7% at the end of microcosm B. By contrast, Bacteroidetes increased more in microcosm M, with the relative abundance being from 1.2% to 25.3%. Proteobacteria showed the opposite trends. The relative abundance of the two phyla had no obvious changes in microcosm R. Moreover, Actinobacteria, Chloroflexi, and Cyanobacteria could be ignored (<1%) with the experimental time. At the genus level, *Marinomonas* (8.2–11.7%) and *Pseudoalteromonas* (74.8–86.6%) were the dominant genera in the three microcosms on day 1, while their abundances varied among the different microcosms. As the experiment progressed, the abundance of *Vibrio* increased rapidly, while that of *Pseudoalteromonas* decreased continuously in three microcosms. On day 5, the dominant genera in microcosm B were *Pseudoalteromonas* (57.2%), followed by *Vibrio* (7.7%), *Pseudophaeobacter* (3.4%), *Marinomonas* (2.8%), and *Pseudochrobactrum* (2.8%), while the dominant genera in microcosm M belonged to *Pseudoalteromonas* (25.7%), *Vibrio* (20.9%), *Loktanella* (5.4%), and *Muricauda* (3.8%). The abundance of *Pseudoalteromonas* in microcosm M (25.7%) was obviously lower than that in microcosm B (57.2%) but higher than that in microcosm R (10.4%), and *Vibrio* exhibited the opposite trend. This indicates that the inclusion of ballast water could enrich *Pseudoalteromonas* and reduce *Vibrio* in the receiving water. In all, it is clear that ballast water could cause changes in the microbial community in receiving water.

### 2.4. Relationship Between Bacterial Communities, MGEs, and ARGs in Microcosms

**Figure 4:**
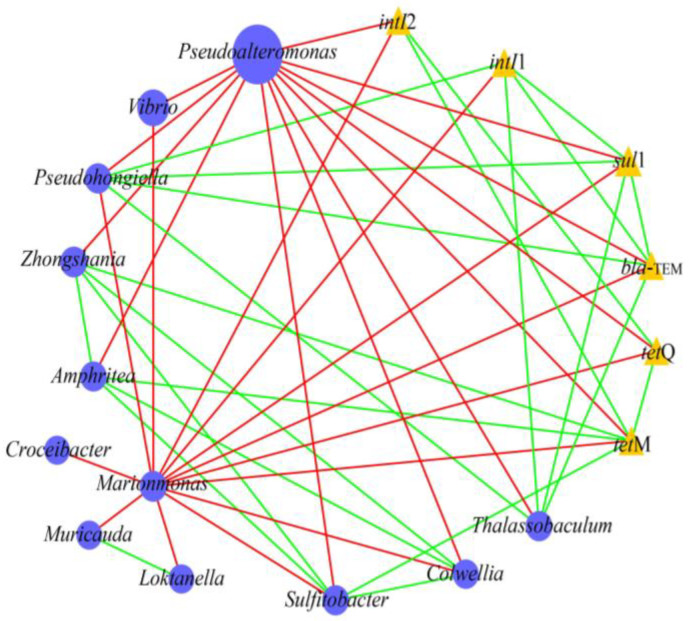
Network analysis showing the potential bacterial hosts for ARGs and MGEs. The square and triangle nodes indicate bacteria and target ARGs/MGEs, respectively. The node size represents the abundance of bacteria/ARGs/MGEs. The edges correspond to significant positive (green) and negative (red) correlations between nodes.

**Figure 5:**
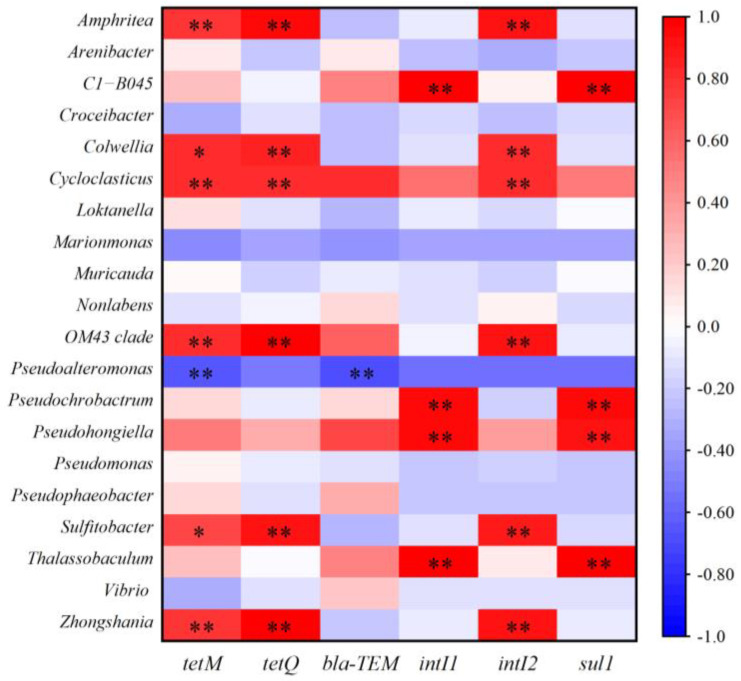
Pearson correlation between ARGs and bacteria genus (* *p* < 0.05, ** *p* < 0.01).

Previous studies reported that the propagation or attenuation of ARGs was closely related to the changes in bacterial community and MGEs [37,38]. Network analysis was performed to investigate the co-occurrence patterns among ARGs, MGEs, and bacterial communities (genus level). As shown in Figure 4, *Pseudohongiella* and *Thalassobaculum* were significantly positively correlated with *sul*1, *bla*_TEM,_ and *intI*1. *Amphritea*, *Sulfitobacter,* and *Zhongshania* were significantly positively related to *tetM*. This indicates that these bacteria were potential hosts of the target ARGs/MGEs. Pearson correlation analysis also supported that these bacteria were closely correlated with ARGs/MGEs (Figure 5). In addition, *Amphritea*, *Colwellia*, *Sulfitobacter*, *Zhongshania*, etc., were positively correlated with *intI*2, *tet*M, and *tet*Q. The co-occurrence of ARGs and MGEs in these bacteria implied that ARGs were likely to be enriched by horizontal transfer after the discharge of ballast water. On the other hand, the changes in the abundance of these potential bacterial hosts might be responsible for the variation in ARGs [27]. For example, the abundances of *Amphritea* were 0.4%, 0.036%, and 0.006% in microcosms B, M, and R on day 5, respectively, indicating that the introduction of ballast water increased its abundance and thus might lead to the enrichment of *tet*M. In conclusion, when ballast water was discharged into the receiving water, some ARGs were probably transmitted and enriched through horizontal gene transfer or the prevalence of bacterial hosts.

## 3. Materials and Methods

### 3.1. Sample Collection and Processing

Ballast water sample was collected from a cargo ship in Yangshan Port (30°37′ N, 122°04′ E) in Shanghai, China. The ballast water was originally pumped in the Arabian Sea (20°22′ N, 72°61′ E), and no exchange was conducted during 28 days of sailing. Sample was collected from the manhole using sterile plastic buckets. Seawater from the East China Sea was collected as the receiving water. The two locations are more than 5000 nautical miles apart and geographically separated by the Indian Ocean and Pacific Ocean, so the water exchange between them is limited. The samples were transported to the laboratory by an ice box within 4 h. The physicochemical characteristics of water samples are given in Table S1.

### 3.2. Metagenomic Analysis of ARGs in the Initial Samples

The ARG profiles in the initial ballast and receiving water samples were determined by Metagenomics. Briefly, 2.0 L of ballast and receiving water samples was filtered by 0.22 μm membrane (Millipore, Billerica, MA, USA), and the DNA in filters was extracted using PowerSoil DNA Isolation Kit (Qiagen, Hilden, Germany). After fragmentation and paired-end fragment library construction, the adaptor-appended fragments were sequenced using the Illumina HiSeq 2000 platform (San Diego, CA, USA). The size of the raw data for each sample was approximately 10 Gb. The low-quality reads that contained ambiguous nucleotides or had a quality score < 20 were removed. Clean reads were assembled into scaftig fragments using MEGAHIT (v1.2.9) assembler [39]. Only scaftig fragments > 500 bp were retained for further analysis. The retained scaftig fragments were used for ORF prediction using MetaGeneMark (v3.38) with default parameters [40]. Non-redundant genes were obtained by CD-HIT (v4.8.1), and SOAPaligner (v2.21) was used to compare the clean reads with the gene pool in order to calculate the number of matches [41]. The unigene was obtained by removing gene classes containing less than three mapped reads. The local BLAST program (2.13.0+) was used to compare the unigene with antibiotic resistance genes database (ARDB) at e-value of <10^−5^ to annotate the ARGs [41].

### 3.3. Microcosm Setup and DNA Extraction

The ballast and receiving water samples were supplemented with 2% (*v*:*v*) 2216E medium before the experiment [27]. Experiments were conducted in 2 L glass jars with a working volume of 1.5 L. Three microcosms were set up: the mixture of ballast water and receiving water at a ratio of 1:1 (microcosm M) to simulate the discharge of ballast water into the receiving water, and ballast water (microcosm B) and receiving water (microcosm R) as the control. Each microcosm was performed in triplicate with a total of nine glass jars. The microcosms were maintained for 5 days at 28 °C in a shaker. Samples (100 mL) were periodically collected from each glass jar (days 1, 3, and 5) on an ultra-clean worktable (AIRTECH, Tianjin, China). The collected samples (number = 27) were filtered through a sterile 0.22 μm membrane (Millipore, Billerica, MA, USA) for genomic DNA extraction using PowerSoil DNA Isolation Kit (Qiagen, Germany).

### 3.4. Quantification of ARGs

The concentration and purity of DNA were evaluated using a microspectrophotometer (Thermo Fisher Scientific, Waltham, MA, USA). The qualified DNA was used in the next analysis. qPCR assays were conducted using the CFX96 Connect System (BioRad, Hercules, CA, USA). The 16S rRNA gene was quantified using a 20 μL volume system, which included 1 μL of template DNA, 1 μL of each primer (10 μM), 7 μL of nuclease-free ddH_2_O, and 10 μL of SYBR Green SuperMix (Zoman, Beijing, China). Four ARGs (*sul*1, *tet*M, *tet*Q, and *bla*_TEM_) and two MGEs (*intI*1 and *intI*2) were determined in 10 μL reaction system, which contained 1 μL of template DNA, 0.5 μL of each primer (10 μM), 3 μL of nuclease-free ddH_2_O, and 5 μL of SYBR Green Mix (Zoman, China). Detailed information regarding the primers is given in Table S2. Ten-fold serially diluted standard plasmid with known quantities was used for the construction of calibration curves. The R^2^ values of calibration curves for all target genes were over 0.99, and the PCR efficiencies were in the range of 0.95~1.15. The abundances of ARGs and MGEs were expressed as copy numbers per bacterial 16S rRNA gene.

### 3.5. Bacterial Community

DNA extracts were subjected to 16S rRNA gene high-throughput sequencing in order to determine the variation in bacterial communities during the experiments. The hypervariable regions (V3–V4) of 16S rRNA gene were amplified with the primers 338F (5′-ACTCCTACGGGAGGCAGCA-3′) and 806R (5′-GGACTACHVGGGTWTCTAAT-3′) containing specific barcodes. PCR was performed in 50 μL reaction system, which consisted of 25 μL of Taq DNA polymerase (5 U/μL), 0.2 μL of each primer (0.5 μmol/L), 4 μL of template DNA, and 20.6 μL of ddH_2_O. The PCR temperature program was initiated with denaturation for 5 min at 95 °C, followed by 30 cycles of 40 s at 95 °C, 40 s at 58 °C, 60 s at 72 °C, and a final elongation for 5 min at 72 °C. The PCR products were sequenced by an Illumina Miseq-PE250 platform. The quantitative insights into microbial ecology (QIIME, v1.8.0) pipeline was used to filter out the low-quality sequences. After chimera detection, the sequences in each library were binned into OTUs at an identity threshold ≥ 97% using USEARCH (v11.0.667). Taxonomic identification of OTUs was annotated against the Greengenes database using the BLAST [42].

### 3.6. Statistical Analysis

A *t*-test was used to assess the significance of the differences in ARGs between microcosms using SPSS 22.0 (*p* < 0.05). Pearson correlation among ARGs/MGEs was also conducted by SPSS 22.0. Network analysis was performed on the basis of Spearman correlation (r > 0.8, *p* < 0.01) between ARGs/MGEs and the dominant genera and then visualized using Cytoscape 3.7.1.

## 4. Conclusions

The spread of ARGs as emerging pollutants in different waters has received widespread attention. The discharge of ballast water leads to the spread of marine organisms and ARGs between geographically separated waters. The main conclusions of this study were as follows: (i) The studied ballast water increased the abundance of ARGs in the receiving water. (ii) MGEs were obviously correlated with ARGs, which might promote the spread of ARGs through horizontal gene transfer. (iii) The bacterial community structure in the receiving water was affected by ballast water. (iv) The discharge of ballast water may affect the potential bacterial hosts of ARGs and ultimately affect the ARGs in receiving waters. In all, this study indicates that shipping can potentially transfer ARGs globally to areas that are relatively unaffected by human activities, thereby leading to the invasion of ARGs. Further research is needed to reveal the fate of ARGs that are introduced by ballast water, especially their spread to the indigenous microbial flora via horizontal gene transfer.

## Figures and Tables

**Table 1:** Pearson’s correlation coefficients between ARGs and MGEs.

	*sul*1	*tet*M	*tet*Q	*bla*_TEM_	*intI*1	*intI*2
***sul*1**	1	/	/	/	/	/
***tet*M**	0.174	1	/	/	/	/
***tet*Q**	0.280	0.972 **	1	/	/	/
***bla*_TEM_**	0.610 *	0.782 **	0.806 **	1	/	/
***intI*1**	0.999 **	0.173	0.277	0.613 *	1	/
***intI*2**	0.247	0.956 **	0.956 **	0.845 **	0.264	1

* Correlation significant at *p* < 0.05; ** Correlation significant at *p* < 0.01.

## Data Availability

Data are contained within the article or Supplementary Materials.

## Supplementary Materials

The following supporting information can be downloaded at https://www.mdpi.com/article/10.3390/antibiotics14040340/s1, Table S1: The physicochemical characteristics of water samples; Table S2: Primers and PCR conditions for ARG analyses; Table S3: Abundance of the ARG subtypes in initial ballast and receiving water; Table S4: The variation in ARG and MGE abundances with the culture time in each microcosm; Figure S1: The relative abundance variation in taxonomic phylum in each microcosm; References [43,44,45,46] were cited by Table S2.
