# Supplementary material for: Simulated Discharge of Ballast Water Reveals Potential Contribution to Spread of Antibiotic Resistance Genes in Geographically Isolated Receiving Waters
Source: Antibiotics (Basel). 2025 Mar 26;14(4):340. doi: 10.3390/antibiotics14040340 (PMC12024036; doi:10.3390/antibiotics14040340)
Supplement: Supplementary file 1 [file antibiotics-14-00340-s001.zip › antibiotics-3538033-supplementary.pdf]

Supplementary information

Simulated discharge of ballast water reveals potential contribution to spread of antibiotic resistance genes in geographically isolated receiving waters

Jianhong Shi [1]*, Chengyuan Ji [1], Rui Wang [2,3], Chaoli Sun [1], Baoyi Lv [1]

[1] *College of Ocean Science and Engineering, Shanghai Maritime University, Shanghai 201306, China*

[2] *CCCC National Engineering Research Center of Dredging and Equipment Co., Ltd., Shanghai, 200082, China*

[3] *Key Laboratory of Dredging Technology, CCCC, Shanghai, 200082, China*

*Corresponding author: Jianhong Shi, PhD
College of Ocean Science and Engineering
Shanghai Maritime University
1550 Haigang Ave, 201306 Shanghai, China
Telephone: (+86) 021-38282518
E-mail: shijh@shmtu.edu.cn

The Supporting materials has including 3 Table and 1 Figure.

Table S1 The physicochemical characteristics of water samples.

Water samples	pH	Salinity	TOC (mg/L)	NO_3^{-}-N (mg/L)	NH_4^{+}-N (mg/L)	PO_4^{3-} (mg/L)
Ballast water	8.3	33.8	2.46	0.0669	0.0237	0.0048
Receiving water	8.5	30.6	2.8	0.1310	0.0241	0.0128

Table S2 Primers and PCR conditions for ARG analyses

Gene	Primer	Sequences	Amplicon size (bp)	Annealing (ºC)	Reference
***sul*1**	forward	CACCGGAAACATCGCTGCA	172	55	1
	reverse	AAGTTCCGCCGCAAGGCT			
***intI*1**	forward	GGCTTCGTGATGCCTGCTT	190	57	
	reverse	CATTCCTGGCCGTGGTTCT			
***intI*2**	forward	GTTATTTTATTGCTGGGATTAGGC	143	57	
	reverse	TTTTACGCTGCTGTATGGTGC			
$\boldsymbol{bla_{TEM}}$	forward	TCGGGGAAATGTGCG	153	60	2
	reverse	GGAATAAGGGCGACA			
***tet*Q**	forward	AGAATCTGCTGTTTGCCAGTG	196	60	3
	reverse	CGGAGTGTCAATGATATTGCA			
***tet*M**	forward	CCGTTGGGAAGTGGAATGC	205	56	
	reverse	TCCGAAAATCTGCTGGGGTA			
**16S rRNA**	forward	GTGCCAGCMGCCGCGGTAA	161	58	4
	reverse	CCGTCAATTCMTTTRAGTTT			

Table S3 Abundance of the ARG subtypes in initial ballast and receiving water

ARGs	Ballast	Receiving
AAC(2')-ic	0.38123	0
AAC(3')-ia	0.37171	0
AAC(3')-iv	0.12387	0.27235
AAC(6')-ib	0.79017	0.77678
AcrA	0	0.54689
AcrB	0.2553	1.02508
ANT(2″)-ib	0.1789	0
ANT(3″)-ib	0.56049	0
arnA	0.2201	0
AAC(3')-ia	0.37171	0
bla-TEM	1.34456	0.69045
bl1-FOX	0.10626	0
bl2b-TEM1	0.17596	0
bl2d-OXA2	0.70123	0
bl3-SHW	0	0.61958
ceoB	0.06029	0.59866
dfrA26	0	0.63453
fosX	0.97545	0.61129
ksgA	0.28752	0.84744
macB	0.03201	0.66679
mexA	0.61169	0
mexB	0.30622	0.02891
mexD	0.12786	0
mexE	0.49497	0
mexF	0.86732	1.17629
mexI	0	0.36402
mexW	0.69216	1.33
mexY	0	0.29988
mfpA	0.29992	0
norm	0	0.34543
oleB	0.17758	0.26273
oprM	0.40503	0
oprN	0.23085	0
pur8	0.27602	0
rosA	0.60955	0
rosB	0.47482	0
smeD	0.0222	0
srmB	0.10992	0
sul1	1.03272	1.02876
sul2	0.24112	0
tet38	0.14752	0
tetG	0.17629	0

tetM	0.47376	0.38168
tetQ	0.44077	0.24681
tetT	0.1713	0
tetV	0.28388	0
tlrC	0.48005	0
vanRC	0	0.66193
vatB	0	0.20646
vatE	0.85251	0.05227
vgaA	0	0.28367

Table S4 The variation in ARG and MGE abundances with the culture time in each microcosm.

	Microcosm B			Microcosm M			Microcosm R		
	Day 1	Day 3	Day5	Day 1	Day 3	Day5	Day 1	Day 3	Day5
*sul*1	3.50±0.20 $\times 10^{-4}$	1.22±0.27 $\times 10^{-3}$	1.20±0.10 $\times 10^{-3}$	1.20±0.13 $\times 10^{-4}$	4.85±0.52 $\times 10^{-4}$	1.64±0.50 $\times 10^{-3}$	1.38±0.27 $\times 10^{-5}$	1.89±0.50 $\times 10^{-4}$	2.93±0.32 $\times 10^{-4}$
tetM	1.09±0.18 $\times 10^{-6}$	1.75±0.58 $\times 10^{-5}$	1.44±0.56 $\times 10^{-5}$	1.65±0.25 $\times 10^{-6}$	5.96±0.42 $\times 10^{-6}$	8.09±0.00 $\times 10^{-6}$	9.90±0.73 $\times 10^{-7}$	3.09±0.15 $\times 10^{-6}$	2.81±0.16 $\times 10^{-6}$
tetQ	2.14±0.68 $\times 10^{-6}$	7.85±0.47 $\times 10^{-6}$	1.33±0.06 $\times 10^{-5}$	3.35±0.11 $\times 10^{-6}$	3.00±0.19 $\times 10^{-6}$	1.33±0.09 $\times 10^{-5}$	2.05±0.26 $\times 10^{6}$	7.26±0.31 $\times 10^{-6}$	1.04±0.07 $\times 10^{-5}$
bla$_{TEM}$	6.52±1.52 $\times 10^{-7}$	3.55±0.47 $\times 10^{-6}$	4.80±0.91 $\times 10^{-6}$	4.22±0.31 $\times 10^{-7}$	1.38±0.37 $\times 10^{-6}$	2.53±0.06 $\times 10^{-6}$	2.16±0.68 $\times 10^{-7}$	1.15±0.12 $\times 10^{-6}$	1.44±0.17 $\times 10^{-6}$
*intI*1	9.15±1.21 $\times 10^{-6}$	2.25±0.26 $\times 10^{-5}$	4.71±1.33 $\times 10^{-5}$	3.24±0.28 $\times 10^{-6}$	2.66±0.89 $\times 10^{-5}$	8.19±1.42 $\times 10^{-5}$	2.96±1.05 $\times 10^{-6}$	2.68±0.19 $\times 10^{-5}$	3.65±0.31 $\times 10^{-5}$
*intI*2	2.63±0.96 $\times 10^{-5}$	2.35±0.40 $\times 10^{-5}$	7.20±0.78 $\times 10^{-5}$	1.01±0.10 $\times 10^{-5}$	2.26±0.23 $\times 10^{-5}$	2.55±0.35 $\times 10^{-5}$	3.02±0.28 $\times 10^{-6}$	1.90±0.09 $\times 10^{-5}$	1.75±0.24 $\times 10^{-5}$

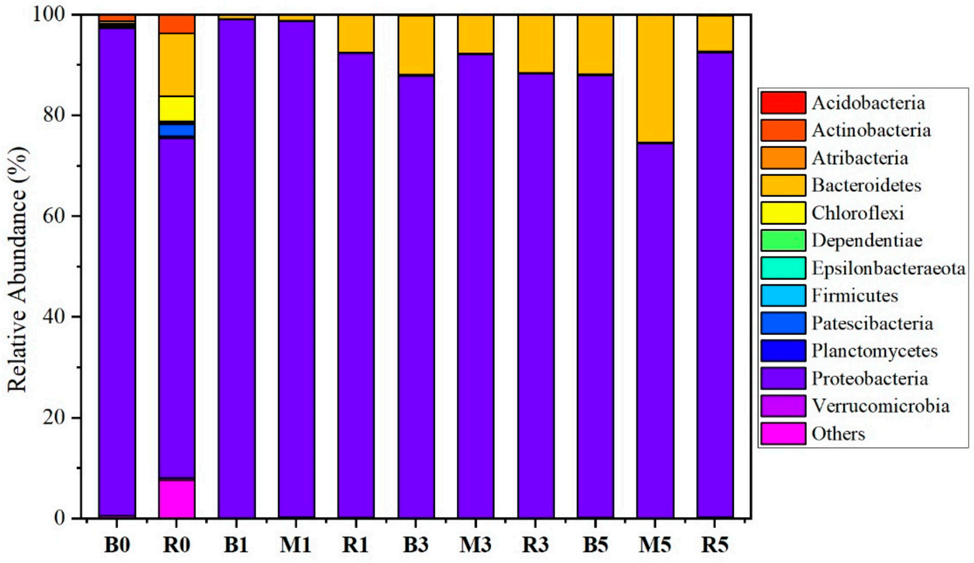

Figure S1. The relative abundance variation of taxonomic phylum in each microcosm.

References

1 Y. Luo, D. Q. Mao, M. Rysz, Q. X. Zhou, H. J. Zhang, L. Xu and P. J. J. Alvarez, Trends in antibiotic resistance genes occurrence in the Haihe River, China, *Environ. Sci. Technol.*, 2010, **44**, 7220–7225.

2 Y. De Gheldre, V. Avesani, C. Berhin, M. Delmée and Y. Glupczynski, Evaluation of Oxoid combination discs for detection of extended-spectrum β-lactamases, *J. Antimicrob. Chemoth.*, 2003, **52**, 591–597.

3 D. Wu, Z. T. Huang, K. Yang, D. Graham and B. Xie, Relationships between antibiotics and antibiotic resistance gene levels in municipal solid waste leachates in Shanghai, China. *Environ. Sci. Technol.*, 2015, **49**, 4122–4128.

4 G. Muyzer, E. C. de Waal and A. G. Uitterlinden, Profiling of complex microbial populations by denaturing gradient gel electrophoresis analysis of polymerase chain reaction-amplified genes coding for 16S rRNA. *Appl. Environ. Microb.*, 1993, **59**, 695–700.
